# Self-Medication as Adaptive Plasticity: Increased Ingestion of Plant Toxins by Parasitized Caterpillars

**DOI:** 10.1371/journal.pone.0004796

**Published:** 2009-03-10

**Authors:** Michael S. Singer, Kevi C. Mace, Elizabeth A. Bernays

**Affiliations:** 1 Department of Biology, Wesleyan University, Middletown, Connecticut, United States of America; 2 Department of Entomology, University of Arizona, Tucson, Arizona, United States of America; University of Birmingham, United Kingdom

## Abstract

Self-medication is a specific therapeutic behavioral change in response to disease or parasitism. The empirical literature on self-medication has so far focused entirely on identifying cases of self-medication in which particular behaviors are linked to therapeutic outcomes. In this study, we frame self-medication in the broader realm of adaptive plasticity, which provides several testable predictions for verifying self-medication and advancing its conceptual significance. First, self-medication behavior should improve the fitness of animals infected by parasites or pathogens. Second, self-medication behavior in the absence of infection should decrease fitness. Third, infection should induce self-medication behavior. The few rigorous studies of self-medication in non-human animals have not used this theoretical framework and thus have not tested fitness costs of self-medication in the absence of disease or parasitism. Here we use manipulative experiments to test these predictions with the foraging behavior of woolly bear caterpillars (*Grammia incorrupta*; Lepidoptera: Arctiidae) in response to their lethal endoparasites (tachinid flies). Our experiments show that the ingestion of plant toxins called pyrrolizidine alkaloids improves the survival of parasitized caterpillars by conferring resistance against tachinid flies. Consistent with theoretical prediction, excessive ingestion of these toxins reduces the survival of unparasitized caterpillars. Parasitized caterpillars are more likely than unparasitized caterpillars to specifically ingest large amounts of pyrrolizidine alkaloids. This case challenges the conventional view that self-medication behavior is restricted to animals with advanced cognitive abilities, such as primates, and empowers the science of self-medication by placing it in the domain of adaptive plasticity theory.

## Introduction

Self-medication is a specific therapeutic and adaptive change in behavior in response to disease or parasitism. Infected animals, for example, could alter their foraging to include medicinal substances in their diets. We view self-medication as a type of adaptive plasticity, which is generally characterized by environmentally induced changes in behavior or phenotype during an individual's lifetime that improve its prospects for survival and reproduction. Adaptive plasticity is specifically expected when there is a predictable trade-off in the adaptive value of alternative phenotypes under detectably different ecological circumstances. Therefore, we expect animals to engage in self-medication when it is adaptive in the presence of disease or parasitism, but not to engage in such behavior in the absence of disease or parasitism due to its fitness cost [1].

Following Janzen's [2] suggestion that vertebrate herbivores might benefit medicinally from the secondary metabolites in their plant food, the empirical study of non-human self-medication has mainly focused on herbivorous and omnivorous vertebrates, such as primates and birds, and their disease-causing parasites. It is probable that many more species of herbivores and omnivores will eventually be found to engage in self-medication behavior [3]–[7]. Some obvious foraging behaviors such as leaf-swallowing by chimpanzees [8], collecting foliage or resin as nest material by birds and ants [9]–[11], and the consumption of dirt or clay (geophagy) [e.g., 12] have been shown to have a medicinal function. However, it is possible that less distinctive behaviors can serve the same function, but have not been recognized in this capacity. Circumstantial evidence has accumulated for the hypothesis that many animal species practice self-medication, yet in most cases definitive tests are lacking [6], [7], [13].

The few experimentally verified cases of self-medication support the theoretical expectation that, when ill from infection or ingested toxins, animals can and do make specific foraging decisions that function specifically to remediate illness. For example, chimpanzees with high intestinal loads of parasitic worms engage in distinctive leaf-swallowing behavior, which is rarely exhibited by healthy chimps [8], [14]. Swallowing the rough, hispid leaves of *Aspilia* plants functions medicinally by dislodging parasites from the gut [15], [16]. Since the original studies of wild chimpanzees exhibiting this rare behavior, evidence has accumulated indicating that all closely observed populations of great apes engage in leaf-swallowing behavior, each population using locally available and chemically disparate plant species with rough, hispid leaves [4], [5]. Similarly, Villalba et al. [17] have recently shown that sheep conditioned with several foods known to cause malaise learned to prefer particular foods containing medicines specific for their illness.

Our study is the first mechanistic demonstration of therapeutic self-medication in an invertebrate animal, and the first to experimentally evaluate self-medication in the context of adaptive plasticity theory, enabled by specific qualities of our study system. *Grammia incorrupta* ( = *geneura*) caterpillars are broad generalist grazers that preferentially ingest non-nutritive plant compounds called pyrrolizidine alkaloids (PA) from certain highly acceptable host-plant species [18]. The preferential ingestion of non-nutritive chemicals is known as pharmacophagy [19]. Once ingested, these compounds are sequestered in the blood and integument of the caterpillars [20], [21]. Previous study of *G. incorrupta* caterpillars also showed that a diet including PA-containing plants improved the survival of field-collected caterpillars by reducing their mortality from parasites. However the PA-plant diet also reduced the growth efficiency of caterpillars [22], suggesting the kind of fitness trade-off that can select for adaptive plasticity. The natural parasites of *G. incorrupta* are insect parasitoids (Tachinidae, Braconidae, and Ichneumonidae) [23], which lay eggs on or in caterpillar hosts, feed and develop as larvae inside their hosts, then emerge to pupate, leaving the host dead. In each of these host-parasitoid interactions, three outcomes have been observed in this system: parasitoid survival and host death, host survival and parasitoid death ( = host resistance), or host and parasitoid death. Extensive study of parasitism of *G. incorrupta* in nature showed three species to cause the most mortality: *Carcelia reclinata* (Tachinidae), *Cotesia* nr. *phobetri* (Braconidae), and *Exorista mella* (Tachinidae) [23].

In the present study, we conducted three experiments. The first tested explicitly the predictions that dietary PA increases the fitness of parasitized caterpillars and reduces the fitness of unparasitized caterpillars (survival and resistance experiment). We quantified the survival of both parasitized and unparasitized caterpillars on synthetic diets that either contained or lacked PA. To additionally test the expectation that *G. incorrupta* caterpillars would increase their PA intake in response to parasitism, we conducted two behavioral experiments comparing PA consumption by parasitized and unparasitized caterpillars. In the feeding choice experiment, we manipulated initial parasitism of caterpillars and observed their subsequent selection and intake of PA and food, offered simultaneously in separate blocks of synthetic media. The no-choice feeding experiment quantified the ingestion of glass fiber discs treated with PA or sucrose, in isolation from other chemicals, by a set of field-collected caterpillars that included naturally parasitized and unparasitized individuals.

## Results

### Survival and resistance experiment

**Figure 1:**
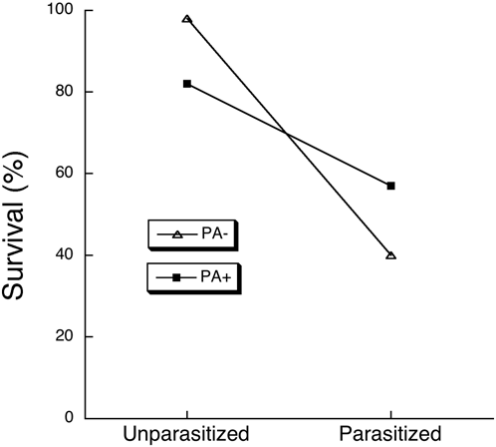
Percentage survival to adulthood of unparasitized and parasitized *G. incorrupta* caterpillars given a synthetic food lacking (PA−) or containing (PA+) 0.1% monocrotaline. Survival of unparasitized caterpillars was significantly higher on PA− food, whereas survival of parasitized caterpillars was significantly higher on PA+ food (see text for statistics).

**Figure 2:**
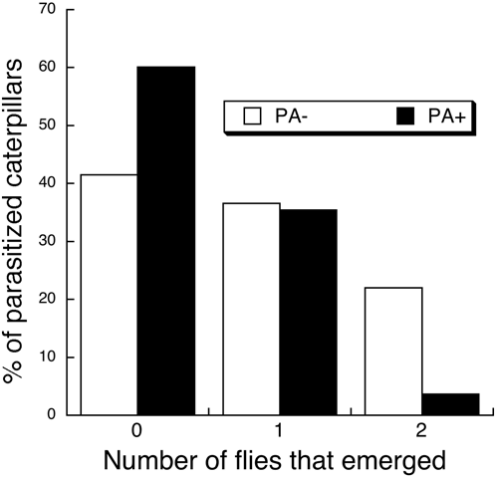
Number of survivors to adulthood of *E. mella* flies that developed in PA+ and PA− caterpillars. Parasitoids had lower survival in PA+ caterpillars (see text for statistics).

The PA+ diet improved the survival of parasitized caterpillars, and decreased the survival of unparasitized caterpillars. Parasitized caterpillars enjoyed a 17% increase in survival on the PA+ diet compared to those on the PA− diet (Fig. 1, Likelihood ratio *χ* ^2^ = 4.92, df = 1, *P* = 0.027). Similar to previous experiments with plants [22], the survival advantage of dietary PA for parasitized caterpillars resulted from an 18% reduction in mortality from parasitoids (Likelihood ratio *χ* ^2^ = 6.80, df = 1, *P* = 0.0091). However, unparasitized caterpillars suffered a 16% reduction in survival on the PA+ diet compared to those on the PA− diet (Fig. 1, Likelihood ratio *χ* ^2^ = 15.85, df = 1, *P*<0.0001). The survival benefit to caterpillars resulted from anti-parasitoid resistance of dietary PA. Flies suffered reduced survival to adulthood in parasitized, PA+ caterpillars versus parasitized, PA− caterpillars (Fig. 2, Likelihood ratio *χ* ^2^ = 14.97, df = 2, *P* = 0.0006).

### Feeding choice experiment

**Figure 3:**
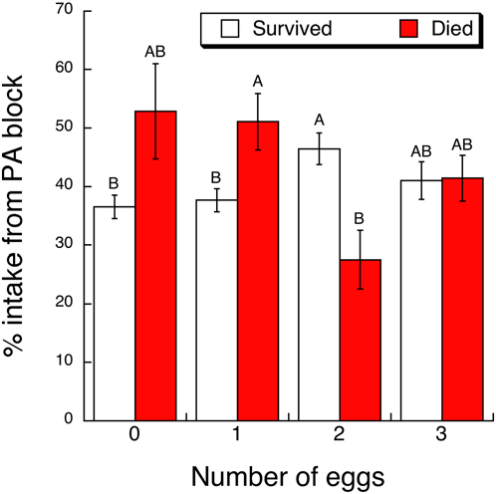
Least square mean (±1 SE) percentage of overall intake from PA block by *G. incorrupta* caterpillars over 5 days in the feeding choice experiment according to parasitism treatment (0–3 *E. mella* eggs) and post-assay survival to adulthood (survived, died). Letters denote significant differences among treatment means from a Tukey-Kramer test (see text for statistics).

The effect of parasitism level (0–3 eggs) on caterpillar food selection and intake was complicated. In apparent contradiction to the self-medication hypothesis, increased levels of parasitism did not statistically increase the percentage of feeding intake from the PA block, or the absolute amount of intake from the PA block (Table 1, Tukey-Kramer tests, α = 0.05). Similarly, increased levels of parasitism did not statistically increase caterpillars' overall intake from PA and food blocks (Table 1, Tukey-Kramer test, α = 0.05). However, some support for the self-medication hypothesis did emerge from analyses that additionally accounted for the survival of each caterpillar to adulthood. Among survivors, caterpillars receiving 2 eggs ate a higher percentage of PA than did caterpillars that received 0 or 1 egg (Table 2, Fig. 3, Tukey-Kramer test, α = 0.05). Among caterpillars that died, the reverse pattern was observed (Fig. 3).

**Table 1:** ANCOVA responses of feeding intake by caterpillars in the choice experiment, quantified in terms of i) the angularly transformed percentage of overall intake from the PA block, ii) the log transformed absolute intake of the PA block, and iii) the log transformed overall intake (PA+food block) over five days.

		% intake from PA block		Amount of PA block eaten		Overall intake	
Factors	df	*F*	*P*	*F*	*P*	*F*	*P*
Parasitism level (P)	3, 99	1.53	0.21	3.39	**0.021**	2.73	**0.048**
Caterpillar family (F)	5, 99	5.81	**<0.001**	23.55	**<0.001**	33.71	**<0.001**
Caterpillar mass (M)	1, 99	1.30	0.26	3.98	**0.049**	1.27	0.26
P×F	15, 99	1.72	0.058	1.91	**0.030**	1.24	0.25
P×M	3, 99	1.10	0.35	3.63	**0.016**	2.60	0.056
F×M	5, 99	1.57	0.17	4.14	**0.0018**	3.66	**0.0044**
P×F×M	15, 99	1.80	**0.045**	1.46	0.14	1.34	0.19

Significant *P* values are marked by boldface type.

**Table 2:** Univariate (ANCOVA) responses of feeding intake by caterpillars in the choice experiment, quantified in terms of i) the angularly transformed percentage of overall intake from the PA block, ii) the log transformed absolute intake of the PA block, and iii) the log transformed absolute intake of the food block over five days.

		% intake from PA block		Amount of PA block eaten		Amount of food block eaten	
Factors	df	F	P	F	P	F	P
Parasitism level (P)	3, 104	1.63	0.19	1.45	0.23	1.99	0.12
Caterpillar family (F)	5, 104	7.36	**<0.001**	9.14	**<0.001**	14.29	**<0.001**
Survival (S)	1, 104	0.60	0.44	0.52	0.47	3.75	0.056
Caterpillar mass (M)	1, 104	0.12	0.73	6.89	**0.010**	10.72	**0.001**
P×F	15, 104	1.71	0.060	1.05	0.41	0.76	0.72
P×S	3, 104	7.98	**<0.001**	3.25	**0.025**	1.13	0.34
P×M	3, 104	1.06	0.37	1.68	0.18	0.98	0.40
F×S	5, 104	1.90	0.10	0.30	0.91	1.64	0.16
F×M	5, 104	0.91	0.48	3.11	**0.012**	2.09	0.073
S×M	1, 104	6.38	**0.013**	0.87	0.35	3.69	0.057

Significant *P* values are marked by boldface type.

**Figure 4:**
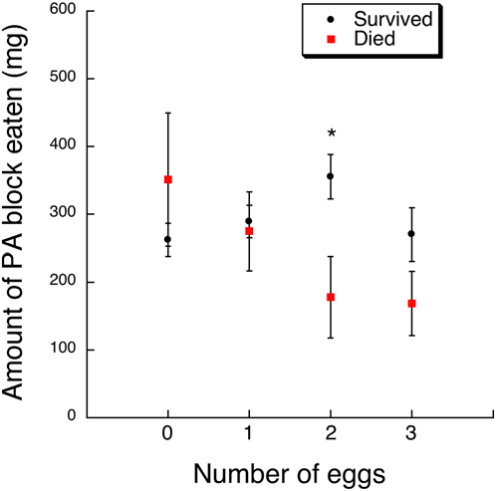
Least square mean (±1 SE) of the total amount of the PA block eaten by *G. incorrupta* caterpillars over 5 days in the feeding choice experiment according to parasitism treatment (0–3 *E. mella* eggs) and post-assay survival to adulthood (survived, died). Asterisks denote significant differences among means of survivors and victims within each treatment from a Tukey-Kramer test (see text for statistics).

**Figure 5:**
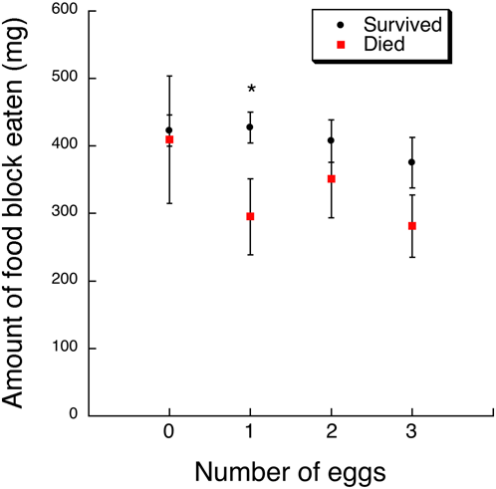
Least square mean (±1 SE) of the total amount of the food block eaten by *G. incorrupta* caterpillars over 5 days in the feeding choice experiment according to parasitism treatment (0–3 *E. mella* eggs) and post-assay survival to adulthood (survived, died). Asterisks denote significant differences among means of survivors and victims within each treatment from a Tukey-Kramer test (see text for statistics).

The feeding choice experiment also showed how absolute PA and food consumption patterns determined caterpillar survival with respect to parasitism levels. In the MANOVA of the absolute consumption of both PA and food blocks, there was a significant interaction between parasitism level and caterpillar survival (Wilks' Lambda *F*_6, 206_ = 3.65, *P* = 0.0018). Increased PA ingestion was evident in survivors that had received 2 parasitoid eggs compared to caterpillars in this treatment that died (Table 2, Fig. 4, Tukey-Kramer test, α = 0.05). Moreover, caterpillars that received 2 parasitoid eggs increased their likelihood of survival via elevated PA ingestion (*χ* ^2^ = 14.72, df = 1, *P* = 0.0001). However, increased PA ingestion in this experiment did not increase the survival of caterpillars that received 0, 1, or 3 parasitoid eggs (Likelihood ratio tests; 0 eggs, *χ* ^2^ = 1.47, df = 1, *P* = 0.22; 1 egg, *χ* ^2^ = 6.93, df = 1, *P* = 0.009 [negative relationship]; 3 eggs, *χ* ^2^ = 1.04, df = 1, *P* = 0.31). The absolute amount of nutritious food ingested during the 5-day feeding period offers some insight into this dilemma (Table 2, Fig. 5). Survivors that had received a single parasitoid egg ate more of the food block than their counterparts that died (Fig. 5). The same non-significant pattern is evident for caterpillars that received 3 parasitoid eggs, but not for those that received 0 or 2 eggs. Indeed, the absolute amount of food ingested increased a caterpillar's likelihood of survival when it received 1 parasitoid egg (*χ* ^2^ = 11.37, df = 1, *P* = 0.0007), but not when it received 0, 2, or 3 parasitoid eggs (Likelihood ratio tests; 0 eggs, *χ* ^2^ = 0.033, df = 1, *P* = 0.85; 2 eggs, *χ* ^2^ = 0.83, df = 1, *P* = 0.36; 3 eggs, *χ* ^2^ = 1.96, df = 1, *P* = 0.16).

The analyses of the total amounts of PA and food ingested by caterpillars are most informative with respect to dietary mechanisms of anti-parasitoid defense. They suggest a multi-faceted feeding response by caterpillars faced with varying magnitudes of parasitoid threat. First, the expectedly high survival of unparasitized caterpillars was not related to their ingestion of PA or food. Second, the survival of singly parasitized caterpillars (30% mortality from parasitoids) was enhanced by greater nutritive intake, suggesting reliance on an immunological response [24]. Third, the substantially lower survival of doubly parasitized caterpillars (47% mortality from parasitoids) was clearly enhanced by greater PA intake, evidence for self-medication, but not by food intake. Finally, the survival of caterpillars initially infected with three parasitoids (52% mortality from parasitoids) was not improved by the amount of nutrients or PA consumed, suggesting that caterpillar defenses were overwhelmed with this level of infection, representing an unusual and extreme case in nature [25].

### No-choice feeding experiment

**Figure 6:**
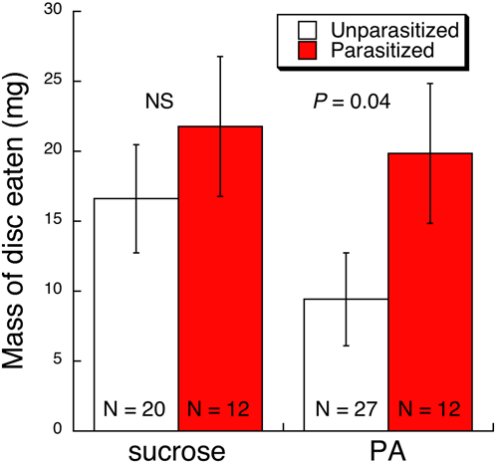
Least square mean (±1 SE) consumption of PA-treated or sucrose-treated glass fiber discs over a 24-h period by field-collected *G. incorrupta* caterpillars according to parasitism status ascertained by post-assay dissection. Parasitized caterpillars ate more of the PA-treated discs than did unparasitized caterpillars; parasitism did not significantly affect consumption of sucrose-treated discs (see text for statistics).

Parasitized caterpillars consumed, on average, 111% more of PA-treated discs during the assay than did unparasitized caterpillars (Fig. 6, Planned contrast *F*_1, 67_ = 4.23, *P* = 0.044). Parasitized caterpillars also consumed, on average, 31% more of sucrose-treated discs than did unparasitized caterpillars, although this difference was not statistically significant (Fig. 6, Planned contrast *F*_1, 67_ = 1.41, *P* = 0.24). These results demonstrate self-medication by showing an increase in PA feeding by parasitized caterpillars in isolation of other chemicals and feeding options. That is, this result dispelled the theoretical possibility that increases in the intake of PA by parasitized caterpillars in the feeding choice experiment came about from an aversion to the food block rather than increased acceptability of the PA block.

## Discussion

Our results demonstrate three essential components of self-medication predicted by adaptive plasticity theory: 1) self-medication behavior improves fitness of animals infected by parasites; 2) self-medication behavior decreases fitness in uninfected animals; and 3) infection induces self-medication behavior.

Predictions 1 and 2 are supported by the survival and resistance experiment and, to a lesser extent, the feeding choice experiment. The expected fitness trade-off of PA ingestion in the presence and absence of parasitism was most clearly seen in the survival and resistance experiment (Fig. 1). In the feeding choice experiment, increased PA ingestion was likewise associated with increased survival in caterpillars that received 2 eggs, whereas the opposite relationship occurred for unparasitized caterpillars. Previous work on this system suggested the existence of this trade-off [22], but the present study shows it directly for the first time. Importantly, these results unambiguously identify PA as an agent of anti-parasitoid resistance for *G. incorrupta*. Although contrary cases exist [e.g., 26], this work complements several previous studies of various caterpillar species showing that the host's ingestion of plant secondary compounds can retard growth and development of its parasitoids [reviewed in 27], [28]; [see also 29], [30]. However, very few studies have shown that such anti-parasitoid effects translate into resistance benefitting host survival, as shown here. The physiological mechanism by which dietary PA confers resistance against parasitoids is not yet known.

Together the feeding choice and no-choice experiments lend support to prediction 3, that parasitism induces self-medication. However, the evidence from the feeding choice experiment is relatively weak, being complicated by extensive variation in PA feeding responses among individuals within parasitism treatments. It is presently unclear why some individual caterpillars exhibited increased PA feeding in response to parasitism while others did not. The no-choice feeding experiment provides more straightforward evidence in support of prediction 3. The presence of infection by parasitoids was clearly associated with increased PA ingestion by caterpillars. We believe the two feeding experiments differed in the variability of PA feeding response in part because of methodological differences in how we scored parasitism in relation to host feeding behavior. In the no-choice experiment, the dissection and scoring of parasitism soon after the feeding assay gave a relatively accurate measure of the effects of parasitoids during the PA feeding assay. By contrast, the caterpillars in the choice experiment received a controlled number of fly eggs in the larval stage before the PA feeding assay, and their parasitoid loads during the feeding assay were unknown. We suspect that many early-stage parasitoids were destroyed prior to the feeding assay by the host encapsulation response, as *G. incorrupta* appears to have an unusually strong encapsulation response [31]. This would have introduced considerable, uncontrolled variation in the parasitoid loads experienced by individual caterpillars during the feeding choice assay.

Taken together, the feeding choice and no-choice experiments show that parasitized caterpillars forage differently than unparasitized caterpillars. One aspect of this foraging difference is an adaptive increase in PA ingestion by caterpillars facing a high threat of mortality from parasitism. Whether the threat of mortality reflects a parasitoid dose-dependent effect (i.e., the number of parasitoid larvae in a host), variation in the developmental stage of individual parasitoids (i.e., early vs. late instars of parasitoid larvae in host), or both is not clear from these experiments.

General observations suggest it is likely that other plant-feeding insect species engage in self-medication because of the ubiquity of dietary chemical defenses [32], and the substantial frequency of parasitism [33], [34] among herbivorous insects. Moreover, many herbivorous insects exhibit various forms of adaptive plasticity [35]. Even herbivores with specialized diets might alter their intake of plant tissue types of varying defensive value in response to parasitism or disease. There exists one other published account of possible self-medication by an herbivorous insect. Parasitized *Platyprepia virginalis* caterpillars (Arctiidae) increased their likelihood of survival by feeding on poison hemlock plants, and parasitized caterpillars preferred poison hemlock over bush lupine, unlike unparasitized caterpillars [36]. Interestingly, this case involved tolerance to (rather than resistance against) parasitoids by host caterpillars, as both host and parasitoid survived in numerous instances. This study is an ambiguous case of self-medication because it is unclear to what extent the results might be due to the parasitoid adaptively manipulating host behavior, as both parasitoid and host benefit from the change in host behavior. Other foraging behaviors in insects have been shown to function as defenses against parasites (e.g., resin-collecting by ants [37]), but none of these other examples shows an adaptive change in behavior in response to infection by parasites.

Self-medication by *G. incorrupta* is distinct from well-understood cases of self-medication in vertebrates by showing a quantitative rather than qualitative change in behavior. That is, parasitism can cause an increase in PA-pharmacophagy, a routine behavior for unparasitized caterpillars. Sick chimpanzees, by contrast, do not typically engage in leaf-swallowing or another specific self-medicative behavior, bitter pith-chewing, in the absence of stress caused by parasites [4], [8]. Self-medication based on a quantitative behavioral change, as seen for *G. incorrupta*, does not easily distinguish itself from routine foraging behavior in observations of wild animals [13]. Consequently, other existing cases of self-medication might be easily overlooked, with behavioral extremes attributed to random variation even for closely observed animals.

We argue that self-medication by *G. incorrupta* is functionally, if not mechanistically, congruent with cases of self-medication by vertebrates. In the vertebrate literature, self-medication has been given the name zoopharmacognosy [38]. The original definition of zoopharmacognosy is “the process by which wild animals select and use specific plants with medicinal properties for the treatment and prevention of disease” [38], broadly encompassing a variety of possible mechanisms such as adaptive plasticity (self-medication as defined here) and prophylaxis (which includes pharmacophagy per se). The role of associative learning in self-medication is a further important mechanistic distinction, as some authors have assumed that associative learning is an essential component of self-medication [6]. Clear experimental proof of self-medication via individual learning was recently demonstrated in domesticated sheep, which learned to ingest particular chemicals that countered toxicity from experimentally applied dietary toxins [17]. Self-medication via social learning is exemplified by wild chimpanzees, which can learn from other individuals' leaf-swallowing behavior to alleviate infection by intestinal nematodes [39]. Among insects such as caterpillars, however, self-medication behavior need not be learned. A previous study of *G. incorrupta* and the related caterpillar *Estigmene acrea* showed that the phagostimulatory taste responses to PA differed between parasitized and unparasitized caterpillars [40]. The gustatory cells of parasitized caterpillars fired action potentials more rapidly than those of unparasitized caterpillars in response to PA, but did not differ in their response to sucrose (a non-medicative feeding stimulant). This specific change in gustation in parasitized caterpillars implies that self-medication in *G. incorrupta* is mediated through plasticity in the peripheral nervous system, without the necessity of associative learning. We hypothesize that parasitized caterpillars can immunologically recognize the presence of internal parasites, and chemically signal the taste system, thus adaptively altering their taste and feeding responses to PA in isolation (as shown here) or in the context of natural host plants. The plausibility of these mechanisms rests on extensive evidence that caterpillars and other insects can immunologically recognize the presence of internal parasites [41], and that chemical feedbacks from the blood to the insect taste system can adaptively alter taste and feeding responses to macronutrients [42].

A functional alliance of self-medication with other forms of adaptive plasticity reinforces the potential importance of self-medication in the ecology, evolution, and conservation of species interactions. A surge of recent publications by many different authors emphasizes the profound consequences of adaptively plastic responses of individuals for understanding population, community, and evolutionary dynamics [43]–[47]. As individuals adaptively alter their behavior and phenotypic traits in response to their ecological circumstances, they can and do alter the demographic outcomes of trophic, competitive, and mutualistic interactions among species [48]. The ecological, evolutionary, and conservation consequences of self-medication are virtually unstudied, despite increasing environmental stresses faced by some of the species, such as great apes, known to self-medicate [4].

In conclusion, our demonstration of self-medication through a shift in the extent of pharmacophagy by *G. incorrupta* caterpillars points to the possibility that more animal taxa than previously believed self-medicate and that known behavioral and physiological mechanisms can mediate self-medication even without associative learning. Our support for self-medication by *G. incorrupta* as a form of adaptive plasticity places the science of self-medication by non-human animals in a theoretical context with broad but relatively unstudied implications for ecology, evolution, and conservation of species interactions.

## Materials and Methods

### Survival and resistance experiment

We tested the survival of caterpillars with a fully factorial manipulation of the presence or absence of parasitism and dietary PA. We used a captive colony of *Exorista mella* flies as experimental parasites. In nature, *E. mella* typically deposits one or two eggs per *G. incorrupta* caterpillar; it is rare to find caterpillars with more than two *E. mella* eggs [25]. At the beginning of the penultimate caterpillar stadium, we experimentally parasitized half of the caterpillars within each dietary treatment. Each caterpillar received two eggs from an individual female fly. We compared the survival of both parasitized and unparasitized caterpillars given a nutritious synthetic food containing 0.1% PA (PA+) or lacking PA (PA-) during the penultimate and ultimate larval stadia. The PA concentration in the PA+ food is in the middle of the range of PA concentrations in natural host plants of *G. incorrupta* (0.0074–1.1%) [20]. This experiment had a fully factorial design to test the effects of diet (PA−, PA+) and parasitism (Para−, Para+) on caterpillar survival and resistance against *E. mella*. All caterpillars were reared in 162.7 ml clear plastic SOLO brand soufflé cups and received a nutritionally balanced, standard rearing diet [49] until they were put onto experimental treatments. Fourteen families were taken from the *G. incorrupta* laboratory culture as second and third instars for inclusion in the experiment. At the start of the sixth (penultimate) larval stadium, each caterpillar was put into its own individual cup and haphazardly assigned to a treatment (PA−/Para−, PA−/Para+, PA+/Para−, PA+/Para+). Four caterpillars from the same family were introduced into the experiment at the same time, one in each treatment. Caterpillars in parasitized treatments (Para+) were placed individually in a container with a single female *E. mella* fly. Female flies were allowed to place two eggs on each caterpillar before the caterpillar was removed and placed in its own cup with its first exposure to experimental food. The PA− and PA+ foods were identical to the standard rearing diet [49], except the PA+ food additionally contained 0.1% monocrotaline, a representative PA. The experiment was done in two temporally displaced trials (*N* = 44, *N* = 38) following the same protocol. All caterpillars were reared on experimental foods until they either pupated or died. We scored as survivors only the individuals that successfully eclosed as adult moths. Among the dead, only the caterpillars that successfully hosted *E. mella* flies (1 or 2 maggots emerged and adult flies eventually eclosed) were scored as killed by parasitoids. To examine how diet affected caterpillar survival, we used a logistic regression (Likelihood ratio test) [50] with survival (yes or no) as the response variable. The effects in the model included diet, caterpillar family, if the individual was parasitized (yes or no), and the interaction parasitism×diet. Upon finding parasitism×diet to be a significant determinant of caterpillar survival, we compared the survival of unparasitized caterpillars in each diet group in a separate logistic regression analysis from that of the survival of parasitized caterpillars in each diet group (Likelihood ratio tests) [50]. The factors in each logistic regression model included diet and caterpillar family. To evaluate the magnitude of resistance against parasitoids conferred by dietary PA, we used a contingency table analysis (Likelihood ratio test) [50] of the likelihood that the number of flies that emerged from each parasitized caterpillar (0, 1, or 2) was independent of diet (PA− or PA+).

### Feeding choice experiment

This experiment compared the dietary intake of nutrients and PA by unparasitized and parasitized final instar caterpillars over a five-day period, which encompassed the majority of their feeding time. All caterpillars were reared in 162.7 ml clear plastic SOLO brand soufflé cups and received a nutritionally balanced, standard rearing diet [49] until they were put onto experimental treatments. Seven families were taken from the *G. incorrupta* laboratory culture as second and third instars for inclusion in the experiment. At the start of the sixth (penultimate) larval stadium, each caterpillar was put into its own individual cup and haphazardly assigned to a treatment (0, 1, 2, or 3 eggs, *N* = 40). Parasitized caterpillars were experimentally parasitized as described for experiment 1, with all eggs on an individual caterpillar from the same individual fly. Again, we used *E. mella* flies to experimentally parasitize caterpillars at the beginning of the penultimate larval stadium. A set of unparasitized caterpillars of the same age and genetic families served as controls. All experimental caterpillars were reared on the same nutritious synthetic food until they molted to the final larval stadium. Then we gave final instar caterpillars in both treatments the same choice of feeding substrates over a 5-day period: one block of nutritious, synthetic food lacking PA, and one substrate block with a 0.1% PA and indigestible cellulose replacing the macronutrients (digestible carbohydrate and protein) of the food block. The choice between food and PA not only allowed us to precisely quantify possible changes in the caterpillars' PA:food intake in response to parasitism, but also created a conservative test of self-medication, as PA consumption required caterpillars to temporarily sacrifice their macronutrient intake. The nutritious food block contained 22.4% protein (casein), 15.2% digestible carbohydrate (sucrose), 2.2% Wesson's salt mix, 11.5% agar, and 48.5% alpha-cellulose. The PA-containing block contained 0.1% monocrotaline instead of protein and carbohydrate, with their combined mass replaced by additional alpha-cellulose. We measured each caterpillar's daily consumption of each food block for the first five days of feeding. To obtain their initial wet masses, we weighed all food blocks prior to introduction to experimental cups. Food blocks were removed at 24-h intervals and replaced with new, weighed blocks. Blocks removed from experimental cups were dried at 60–70°C to a stable mass (<1% change in mass between successive days). We estimated the dry mass of each fresh block with a conversion curve [51]. After the feeding choice assay, all caterpillars were reared on standard rearing food until they either pupated or died. Survivors and parasitoid victims were scored as described for experiment 1. Caterpillars that did not feed (*N* = 1) or died before or during the feeding choice assay (*N* = 32) were excluded from statistical analyses.

To evaluate evidence for self-medication, we conducted several statistical analyses. First, we compared three different measures of consumption by caterpillars that had been experimentally parasitized at different levels (0–3 eggs). The different response variables (angularly transformed % intake from PA block, log transformed absolute intake of PA block, log transformed overall intake of PA and food blocks) were analyzed separately with ANCOVA models each containing a fully factorial combination of parasitism level, caterpillar family, and caterpillar mass at the beginning of the 7^th^ larval stadium. Because of extensive variation in some parasitism treatment groups appeared to depend on caterpillar survival, we analyzed three different measures of feeding in relation to the survival of caterpillars to adulthood following the feeding assay. The different response variables (angularly transformed % intake from PA block, log transformed absolute intake of PA block, log transformed absolute intake of food block) were analyzed separately with ANCOVA models each containing a factorial combination of parasitism level, caterpillar family, survival to adulthood (yes or no), caterpillar mass at the beginning of the 7^th^ larval stadium, and all 2-way interactions. Because of potential non-independence between measures of absolute PA and food intake, we first analyzed the log transformed masses of PA and food blocks consumed in a MANCOVA model with the following terms: parasitism level (0–3 eggs), caterpillar family, survival to adulthood (yes or no), caterpillar mass at the beginning of the 7^th^ stadium, and all 2-way interactions. To gain greater insight into how diet improves the survival of parasitized caterpillars, we analyzed the likelihood of caterpillar survival with Likelihood ratio tests. The first Likelihood ratio test model included parasitism level (0–3 eggs), total food consumed (log transformed), total PA block consumed (log transformed), caterpillar family, caterpillar weight at the beginning of the 7^th^ stadium, as well as the following interactions: parasitism level×total food consumed, parasitism level×total PA block consumed. Upon finding significant interactions, we ran separate Likelihood ratio tests for caterpillars in each parasitism treatment group. These tests included the same terms in the model except for parasitism level and its interactions.

### No-choice feeding experiment

This experiment was designed to compare precisely the power of PA-feeding stimulation in unparasitized and parasitized caterpillars. One hundred late instar *G. incorrupta* caterpillars were collected from Harshaw Canyon, Patagonia Mountains, Santa Cruz Co., Arizona on 14 April 2005. Most of the field-collected caterpillars were penultimate instars or early final instars. They were brought back to the laboratory and given the standard rearing food for eight days. Eighty final instar caterpillars that appeared to be still feeding were then randomly divided into two no-choice feeding treatments: a glass fiber disc treated with either 0.01 mM monocrotaline (PA) or 1.0 mM sucrose in distilled water. The concentrations of PA and sucrose used here were previously shown to elicit strong responses by gustatory cells in electrophysiological experiments [40]. Each experimental caterpillar was confined to its own closed 162.7 ml clear plastic SOLO brand soufflé cup with the weighed, dry glass fiber disc impaled on a pin pushed through one side of the cup. The entire disc was accessible to each caterpillar. To soften the disc during the feeding assay, a piece of moist cotton was placed on the floor of the cup on the opposite side of the pushpin. Caterpillars were left to feed on the discs for 24 h at room temperature (23°C), at which point all discs were removed, dried for 24 h, then reweighed. Following the feeding assay, the caterpillars were dissected to identify individuals harboring larval parasitoids. All caterpillars scored as parasitized contained one or more third (final) instar tachinid fly larvae. The specific identities of these tachinid larvae could not be determined. However, the appearance, timing, and frequency all suggested that most or all of them were *Carcelia reclinata*. To calculate the mass of each disc consumed during the assay (amount eaten), the final dry mass of each glass fiber disc was subtracted from its initial dry mass. We analyzed the amount eaten (log-transformed) as a response variable in a general linear model [50] with feeding treatment (PA or sucrose), parasitism (yes or no) and diet×parasitism as factors. We used separate planned contrasts to compare the amount eaten by unparasitized and parasitized caterpillars in each of the feeding treatment groups. We excluded from the analysis caterpillars that consumed no measurable amount of their disc on the basis that such individuals were no longer in the feeding stage of their larval period.
